# Sequential host-bacteria and bacteria-bacteria interactions determine the microbiome establishment of *Nematostella vectensis*

**DOI:** 10.1186/s40168-023-01701-z

**Published:** 2023-11-18

**Authors:** H. Domin, J. Zimmermann, J. Taubenheim, G. Fuentes Reyes, L. Saueressig, D. Prasse, M. Höppner, R. A. Schmitz, U. Hentschel, C. Kaleta, S. Fraune

**Affiliations:** 1 Institute for Zoology and Organismic Interactions, Heinrich-Heine-University Düsseldorf, Düsseldorf, 40225 Germany; 2 Research Group Medical Systems Biology, Institute of Experimental Medicine, Christian-Albrechts-University Kiel, Kiel, 24105 Germany; 3 Institute for General Microbiology, Christian-Albrechts-University Kiel, Kiel, 24105 Germany; 4 Institute for Clinical Molecular Biology, Christian-Albrechts-University Kiel, Kiel, 24105 Germany; 5 RD3 Marine Symbioses, GEOMAR Helmholtz Centre for Ocean Research, Kiel, 24105 Germany; 6 Christian-Albrechts-University Kiel, Kiel, 24105 Germany

## Abstract

**Background:**

The microbiota of multicellular organisms undergoes considerable changes during host ontogeny but the general mechanisms that control community assembly and succession are poorly understood. Here, we use bacterial recolonization experiments in *Nematostella vectensis* as a model to understand general mechanisms determining bacterial establishment and succession. We compared the dynamic establishment of the microbiome on the germfree host and on inert silicone tubes.

**Results:**

Following the dynamic reconstruction of microbial communities on both substrates, we show that the initial colonization events are strongly influenced by the host but not by the silicone tube, while the subsequent bacteria-bacteria interactions are the main driver of bacterial succession. Interestingly, the recolonization pattern on adult hosts resembles the ontogenetic colonization succession. This process occurs independently of the bacterial composition of the inoculum and can be followed at the level of individual bacteria. To identify potential metabolic traits associated with initial colonization success and potential metabolic interactions among bacteria associated with bacterial succession, we reconstructed the metabolic networks of bacterial colonizers based on their genomes. These analyses revealed that bacterial metabolic capabilities reflect the recolonization pattern, and the degradation of chitin might be a selection factor during early recolonization of the animal. Concurrently, transcriptomic analyses revealed that *Nematostella* possesses two chitin synthase genes, one of which is upregulated during early recolonization.

**Conclusions:**

Our results show that early recolonization events are strongly controlled by the host while subsequent colonization depends on metabolic bacteria-bacteria interactions largely independent of host ontogeny.

Video Abstract

**Supplementary Information:**

The online version contains supplementary material available at 10.1186/s40168-023-01701-z.

## Introduction

All multicellular organisms live in association with microbes. These microbes can have a variety of effects and functions in metabolism [1], immunity [2], pathogen resistance [3], development [4], and behavior of their macroscopic host [5]. The growing understanding of the effects of the microbiome on its host raises the questions of how microbial communities assemble, how they resist perturbation and how they function in the context of the host.

In host-microbe research, the complexity of the microbiome poses one of the biggest challenges. To facilitate questions about how beneficial communities assemble, several ecological models were applied to a microbial scale, such as niche theory and neutral theory [6], null models [7], or Vellend’s understanding of community assembly [8]. Nemergut et al. updated Vellend’s framework on community assembly for microbial communities, explaining assembly processes through only four processes: selection, diversification, dispersal and drift, while also accounting for the differences between macrobial and microbial community assembly [8, 9]. While dispersal and drift are considered to be more stochastic and therefore mainly dictated by chance, selection and diversification are considered to be more deterministic and therefore mainly dictated by bacterial, environmental, or host factors that exert selection or diversification pressure.

Recently, *Nematostella vectensis* became popular as a model to understand host-microbe interactions [10]. *Nematostella* is a cnidarian sea anemone belonging to the Anthozoans, and although cnidarians belong to the early-branching metazoans, *Nematostella* exhibits a surprisingly large genetic complexity, possessing most signaling pathways for development and immunity important in bilaterian animals [11, 12]. *Nematostella* readily undergoes its complete life cycle under laboratory conditions and its whole bacterial community composition was characterized over the course of its ontogeny, as well as its virome [13, 14]. Thereby, the microbiome of *Nematostella* changes with developmental age, shows spatial structuring along the body column, and exhibits a diurnal pattern [14–17]. In doing so, it shows strong resistance to community overgrowth by one member [18]. Recently, high microbial plasticity in response to environmental changes has been functionally linked to thermal adaptation in *Nematostella* [19].

Here, we aim to understand the fundamental principles underlying the establishment and succession of complex microbial consortia on host tissue. Our results show that the recolonization dynamics recapitulate an ontogenetic colonization pattern of *Nematostella*, regardless of the initial composition of the inocula. Thereby, single members of the microbiome can be divided into early- and late-colonizing bacteria, which are defined by their appearance during recolonization. Early recolonization correlated with a high abundance of polysaccharide degradation pathways, especially for potentially host-provided chitin. In agreement, transcriptomics analysis showed an increased expression of host chitin synthase genes. In contrast, late-appearing bacteria were increasingly capable of oxidizing compounds such as nitrite and sulfide which earlier colonizers potentially released by nitrate and sulfate reduction. Thus, we highlight the successive nature of bacterial colonization of the host *Nematostella* and suggest a role for host-microbe interactions via chitin as a driver of early recolonization events and bacteria-bacteria interactions as a driver of later colonization events.

## Materials and methods

### Animal culture

The adult animals of the laboratory culture were F1 offspring of CH2XCH6 individuals collected from the Rhode River in Maryland, United States [20, 21]. Animals were kept under constant, artificial conditions without substrate or light. For *Nematostella* Medium (NM), Red Sea Salt was diluted in Millipore H_2_O and adjusted to 18 °C and 16‰ salinity. Feeding occurred 2–3 times a week with first instar nauplius larvae of *Artemia salina* (Ocean Nutrition Micro Artemia Cysts 430–3500 g, Coralsands, Wiesbaden, Germany). Primary polyps were fed with homogenized larvae until they were big enough to feed on whole larvae. Spawning was induced adapted after Genikhovich et al. by shifting the temperature to 25 °C and exposure to light for 10 h [22]. Fertilization was performed in vitro in petri dishes by transferring the egg packages into NM containing sperm. Fertilization of the eggs was performed within 1 h of release of the egg package from the mother.

### Antibiotic treatment

Antibiotic treatment was adapted after Domin & Gutiérrez et al. [18]. Sterility was confirmed firstly via plating of homogenized polyps on marine broth (MB) plates. Absence of CFUs was interpreted as sterile. Secondly, sterility was checked via a PCR with primers specific for V1-V2 region of the bacterial 16S rRNA gene (27F and 338R). Although a slight band could be observed, no recovery of bacteria over the course of the experiment could be observed in subsequent PCRs and plating on MB plates, attributing the slight PCR band to dead bacterial matter.

### Recolonization

For the recolonization experiments of live polyps, the protocol for conventionalized recolonized *Hydra* polyps was modified [3]. The germfree adult polyps were recolonized with the microbiota of three different developmental stages, respectively. For the four time points (2, 7, 14, and 28 days post recolonization), four germfree polyps were pooled in one vessel. Experiments were conducted with five independent replicates. For recolonization with adult stages, one adult polyp per one germfree polyp was homogenized (4 homogenized polyps/ 50 mL NM), for early and juvenile stages approximately 0.1 mL of animals per adult polyp was homogenized (0.4 mL homogenized animals/ 50 mL NM). Early stages were 6 days old, juvenile stages 54 days old. After 24 h, the medium was exchanged to remove tissue debris and non-associated bacteria. After another 24 h, samples for the first time point (2 dpr) were collected. For each sample, one polyp was used. After washing the polyp three times, it got either homogenized in NM for gDNA extraction or frozen in liquid nitrogen for RNA extraction. If the animals were homogenized in NM, 1/100 of the homogenized animals was plated on marine broth plates prior to gDNA extraction and the plates were incubated for at least 2 days at 18 °C to count CFUs. Due to extraction difficulties, the experiments for gDNA extraction and RNA extraction were performed separately. The polyps were not fed for the whole duration of the antibiotic treatment and the recolonization experiment.

For the recolonization of silicone tubes, hollow silicone tubes with an inner diameter of 3 mm, an outer diameter of 5 mm, and a wall thickness of 1 mm were cut into 1-cm-long pieces. Tubes were recolonized and sampled exactly like the adult polyps but with 10% MB in NM. Both recolonizations, polyps and tubes, were conducted separately. For sampling, tubes were washed three times and bisected longitudinally. One half was used for gDNA extraction and 16S rRNA gene sequencing, the other half was used for biofilm quantification with crystal violet. For this, the tubes were incubated in 1 mL of 0.1% crystal violet solution for 15 min. Afterwards, tubes were washed three times with water before the tubes were dried overnight. Then crystal violet was washed off the tubes with 500 µL of 95% ethanol for 15 min with slight agitation. Absorbance was measured at 550 nm.

### DNA extraction and 16S rRNA gene sequencing

Prior to gDNA extraction, the animals were washed three times with 500 µL sterile NM and frozen without liquid at − 20 °C until extraction. The gDNA was extracted with the DNeasy Blood & Tissue Kit (Qiagen, Hilden, Germany) as described in the manufacturer’s protocol. DNA was eluted in 50 µL elution buffer. The eluate was frozen at − 20 °C until sequencing. For each sample, the hypervariable regions V1 and V2 of bacterial 16S rRNA genes were amplified. The forward primer (5′-AATGATACGGCGACCACCGAGATCTACAC XXXXXXXX TATGGTAATTGT AGAGTTTGATCCTGGCTCAG-3′) and reverse primer (5′-CAAGCAGAAGACGGCATACGAGAT XXXXXXXX AGTCAGTCAGCC TGCTGCCTCCCGTAGGAGT-3′) contained the Illumina Adaptor p5 (forward) and p7 (reverse). Both primers contain a unique 8 base index (index; designated as XXXXXXXX) to tag each PCR product. As positive control, the zymobiomics microbial community standard from Zymo Research was used. The negative control does not contain any template. Sequencing was conducted as described in [18] on the Illumina MiSeq platform with v3 chemistry. The raw data are deposited at the Sequence Read Archive (SRA) and available under the project ID PRJNA902551.

### 16S rRNA gene sequences processing

Filtering and taxonomic analysis were conducted according to the qiime2 pipeline [23, 24]. Sequence quality filtering was performed via DADA2 and taxonomic analysis via the q2-feature-classifier plugin for qiime2 with the Greengenes 13_8 97% OTU data set as reference [25–27]. Further downstream analysis was conducted using the R package phyloseq [28], and plots were generated with the R package ggplot2 [29]. The statistical tests adonis and anosim were calculated with the R package vegan [30]. Because qiime2 creates the abundance table according to amplicon sequence variants (ASVs) and not operational taxonomic units (OTUs) on a specific identity percentage anymore, we manually clustered the ASVs into OTUs with 97% identity with cd-hit-est [31, 32] for the metabolic pathway analysis. The output sequences were called clusters instead of ASV or OTU.

### Quantification of relative bacterial abundance

In order to quantify the relative bacterial abundance in comparison to host tissue, we performed quantitative real-time PCR with the 27F/338R bacterial primers (F 5′-AGAGTTTGATCCTGGCTCAG-3′, R 5′-TGCTGCCTCCCGTAGGAGT-3′), and primers for the elongation factor 1alpha gene (F 5′-GTAGGCCGTGTTGAGACTG-3′, R 5′-CACGCTTGATATCCTTCACAG-3′) of *Nematostella*. The expression levels were calculated according to the ΔΔCT method [33]. We used the GoTaq qPCR Master Mix (Promega) according to the manufacturer’s protocol. Cycling was performed in MicroAmp 0.2 mL optical strips (Applied Biosystems), and a QuantStudio 3 qPCR system (Applied Biosystems).

### RNA extraction and sequencing

Prior to RNA extraction, polyps were washed three times in sterile NM. After pipetting off as much liquid as possible, polyps were immediately frozen in liquid nitrogen and stored at − 80 °C until extraction. Total RNA was extracted with the RNeasy Plant Mini Kit (Qiagen) according to the manufacturer’s protocol. RNA was eluted in 30 µL RNase-free water that got reapplied on the column’s membrane and eluted again. RNA quality was checked via application on an agarose gel and measured on a Qubit. RNA libraries were constructed using the TruSeq stranded mRNA (incl. p-A enrichment) protocol and were sequenced on a HiSeq4000 with a 2 × 75 bp data yield and a paired-end mode. The raw data are deposited at the Sequence Read Archive (SRA) and available under the project ID PRJNA909070.

### RNA sequence analysis

RNA-sequencing reads were adapter and quality trimmed using trimmomatic [34] in paired-end mode using the following options: ILLUMINACLIP:{adapter.fasta}:2:30:10 LEADING:3 TRAILING:3 SLIDINGWINDOW:4:20 MINLEN:36. Trimmed reads were mapped against the Vienna reference *Nematostella* transcriptome using the Bowtie2 software with default parameters [35, 36]. Resulting sam files were converted to bam format using the samtools suite [37]. Read counts per transcript were estimated by the Salmon software package using default parameters and the -l ISR option [38]. Differential analysis of the count data was performed using R and the DESeq2 software package [39, 40]. For differential gene estimation log-fold change shrinkage was performed before testing differences with a Wald-*p*-test (betaPrior = TRUE), all genes where the adjusted *p*-value was lower than *α* = 0.05 were considered differentially expressed, regardless of fold change.

### Bacteria isolation and culturing

Bacteria were isolated of planula larvae, juveniles, and adult polyps. Whole body homogenates were spread out on MB, LB, R2A, and count agar plates. Plates were incubated at 4, 18, 20, 30, or 37 °C. Colonies to pick were selected by their morphology in regard to color, size, and shape to exclude redundancy. The goal was to obtain a library of as many bacteria colonizing *Nematostella vectensis* over the whole life cycle as possible under the given culturing conditions. Purified single colonies were transferred into the respective liquid media and saved as either cryostocks or glycerol stocks (10 or 25% final glycerol concentration). If bacteria were regrown for experiments, it was first tried to culture them in MB at 30 °C to ensure equal growth conditions. All bacteria used for the mono-association experiments were able to grow on MB. Also all bacterial isolates were clustered against the ASVs obtained from the 16S rRNA gene sequencing. All isolates match to an ASV and are indicated so in Additional file 2: Table S1.

### Mono-association experiments

Prior to mono-associations, adult polyps were treated with antibiotics in the same manner as for the recolonization experiments. Bacteria for mono-associations were selected for their succession pattern during the recolonization process. Bacteria were grown overnight, diluted in fresh medium, and grown to an OD600 of 0.1. Each polyp was recolonized with a calculated OD600 of 0.001 (approx. 50,000 cells) of a single bacterial strain in 3 mL and incubated at 18 °C (*n* = 5). After 24 h, the medium was exchanged with fresh sterile medium. Seven days after recolonization, polyps were homogenized and spread out on MB plates. Colonies were counted after 3 days of incubation at 18 °C.

### Heatmap creation

For the heatmap, ASVs were filtered for those ASVs which were constantly detected in at least one of the four different timepoints of recolonization across all samples (minimum relative abundance was 0.005%). Afterwards, the abundance data for each ASV were normalized to range between 0 and 1 for each sub-heatmap (columns). The basis for the ordering of the ASVs in the heatmap was hierarchical clustering with minor manual adjustments for clarity.

### Genome isolation/sequencing/assembly/annotation

Genomic DNA of single bacterial strains was isolated using the Genomic DNA Purification Kit (Promega) using the protocol for gram positive bacteria. Libraries were prepared using the Nextera DNA Flex Kit (Illumina). Sequencing was performed on an Illumina NextSeq 1500 with a read length of 2*150 bp to approximately 60–80X coverage per genome. For assembly, genomic paired-end reads were first trimmed with TrimGalore [41] to remove any remaining adapter sequences and reads shorter than 75 base pairs. Cleaned reads were subsequently assembled into draft genomes with SPAdes [42] and all-default settings. Finally, for each draft assembly, gene models were annotated using Prokka [43] and its built-in reference database. The raw data are deposited and available under the project ID PRJNA912077, the code can be found in a github repository under the following link: https://github.com/ikmb/assembly-bacteria.

### Metabolic pathway analysis

The inference of bacterial metabolic capacities and the comparison of potential pathway abundances over time was done by metabolic pathway analysis. For the prediction of metabolic pathways, gapseq was employed [44]. As input served sequence data from newly assembled genomes as well as published genomes from NCBI (Additional file 2: Table S2). gapseq was run with default parameters (bitscore threshold of 200) with pathway definitions derived from MetaCyc [45]. In addition, other bacterial traits, potentially relevant in host interactions, were inferred using Abricate and the virulence factor database VFDB [46, 47]. The potential pathway abundances were calculated from genomic capacities and bacterial abundance data generated with 16S rRNA gene sequencing. For this means, the relative bacterial abundance for each timepoint, bacterial source, and replicate were computed. Next, for each pathway, the sum of relative abundances from all bacteria which were predicted to possess the corresponding pathway was determined. This resulted in relative cumulative pathway abundances that were used to compare changes in metabolic capacities over time.

Pathways associated with early (2d,7d) and late (14d, 28d) time points were summarized into subsystems. Associated pathways were determined by random forest feature selection using Boruta and the importance score of pathways was summed up for each subsystem. Subsystems with an importance score ≥ 0.5 were shown.

## Results

### Adult polyps control initial colonization events

**Fig. 1:**
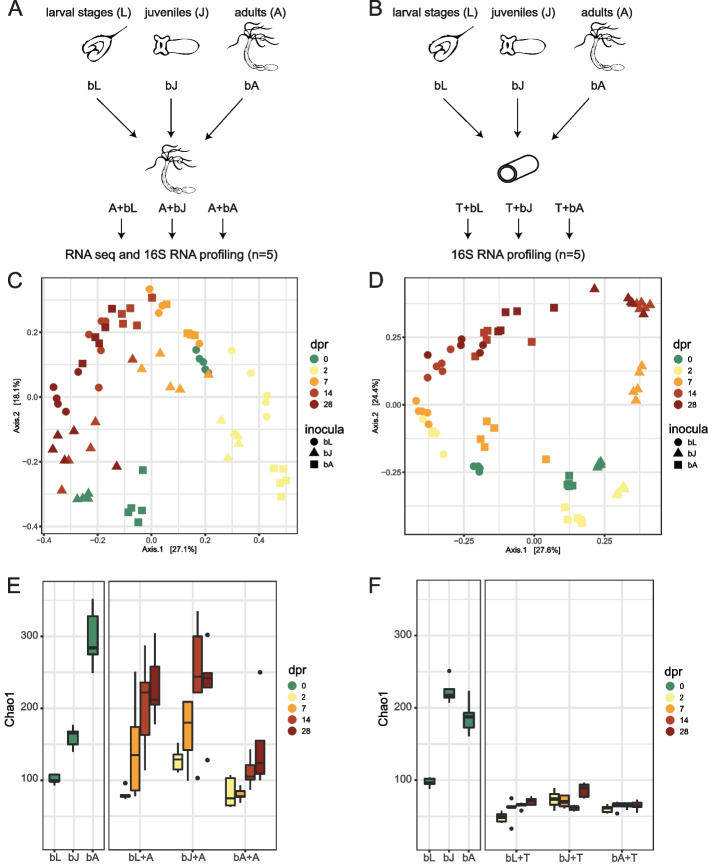
Bacterial recolonization dynamics of germfree polyps and silicone tubes. **A**, **B** Experimental setup for recolonization of **A** adult polyps and **B** silicone tubes. Samples were taken from the inocula and 2, 7, 14, and 28 days post recolonization. **C**, **D** Principal coordinate analysis (PCoA) based on the Bray–Curtis dissimilarity for the recolonization of **C** polyps and **D** tubes. **E**, **F** Chao1 measure for the recolonization experiment of **E** polyps and **F** tubes. The chao1 measure of the inocula is shown on the left side, while the change of the chao1 measure over time is shown on the right. The different time points of the recolonization are color-coded, while the developmental stage from the source of the inoculum is shape-coded. bL = bacteria of Larvae, bJ = bacteria of Juveniles, bA = bacteria of Adults, dpr = days post recolonization

In order to understand the rules underlying the establishment of complex microbial consortia on host tissue, we performed a comparative recolonization experiment on host tissue and inert silicon tubes (Fig. 1A, B, Additional file 2: Table S3-4). We used adult polyps of the sea anemone *Nematostella vectensis* that were depleted of their microbiome, and sterile silicone tubes to imitate an inactive polyp with an inner (gastrodermic) and an outer (ectodermic) surface. Both, antibiotic-treated polyps und sterile tubes, were recolonized with three different bacterial consortia of larvae (bL), juvenile (bJ), and adult polyps (bA), respectively (Fig. 1A, B). The three bacterial inocula differed significantly in their composition (Fig. 1C–F. Additional file 1: Figure S1-S2, Additional file 2: Table S5-S6, pairwise PERMANOVA, pseudo-F value for the polyp experiment: bL-bA 39.94, for bJ-bA 33.99, for bL-bJ 68.52, p and q < 0.05; for the tube experiment: bL-bA 90.83, for bJ-bA 43.53, for bL-bJ 104.75 p and q < 0.05).

A comparison of the bacterial community successions on host tissue and silicone tubes revealed significant differences (Adonis R^2^ 0.14603, *p*-value 0.001; Anosim R 0.4661, *p*-value 0.001, Additional File 1: Figure S3). Considering effect size (R^2^ values, Adonis) and dissimilarity (R values Anosim), days after recolonization (dpr) explained a higher proportion of bacterial variation on the polyp than inocula. In contrast, the recolonization of tubes was influenced similarly by both inoculum and dpr (Table 1). According to the statistics of the polyps, the inocula explain similar amount of differences for all tested weighted metrics. Only two unweighted metrices (unweighted UniFrac and binary Jaccard) explained less variation by dpr. Therefore, we performed Principal Coordinate Analysis (PCoA) based on Bray–Curtis metric to consider community evenness (Fig. 1C, D). Additionally, we calculated distances with weighted and unweighted UniFrac to include phylogenetic information (Additional File 1: Figure S4 and S5).

**Table 1:** Statistical analysis of the influence of the inocula and the days post recolonization on the recolonization dynamics calculated for six different distance metrices. The R value hereby represents a ratio of dissimilarities, while the R^2^ value is a measure of the effect size

Substrate	Parameter	Metric	Adonis R^2^	Adonis p	Anosim R	Anosim p
Polyp	Inocula	Bray–Curtis	0.16	0.001	0.24	0.001
		Jensen-Shannon Divergence	0.21	0.001	0.26	0.001
		Weighted Unifrac	0.13	0.001	0.19	0.001
		Unweighted Unifrac	0.16	0.001	0.35	0.001
		Jaccard	0.13	0.001	0.24	0.001
		Binary Jaccard	0.16	0.001	0.36	0.001
	dpr	Bray–Curtis	0.40	0.001	0.58	0.001
		Jensen-Shannon Divergence	0.52	0.001	0.58	0.001
		Weighted Unifrac	0.42	0.001	0.61	0.001
		Unweighted Unifrac	0.19	0.001	0.28	0.001
		Jaccard	0.31	0.001	0.58	0.001
		Binary Jaccard	0.18	0.001	0.30	0.001
Tube	Inocula	Bray–Curtis	0.32	0.001	0.51	0.001
		Jensen-Shannon Divergence	0.41	0.001	0.54	0.001
		Weighted Unifrac	0.73	0.001	0.40	0.001
		Unweighted Unifrac	0.24	0.001	0.40	0.001
		Jaccard	0.25	0.001	0.51	0.001
		Binary Jaccard	0.30	0.001	0.52	0.001
	dpr	Bray–Curtis	0.41	0.001	0.54	0.001
		Jensen-Shannon Divergence	0.48	0.001	0.51	0.001
		Weighted Unifrac	0.44	0.001	0.37	0.001
		Unweighted Unifrac	0.37	0.001	0.47	0.001
		Jaccard	0.34	0.001	0.54	0.001
		Binary Jaccard	0.31	0.001	0.43	0.001

The PCoA and hierarchical clustering also revealed qualitative differences between host and tube colonization succession (Fig. 1C,D; Additional file 1: Figure S1- S2). During the recolonization of the tubes, initial differences originating from the three different inocula were maintained in the different treatments throughout the experiment (Fig. 1D, Additional file 1: Figure S1). While on principal coordinate 1 (PC1), the differences in the bacterial communities of the inocula became apparent, the bacterial succession in all three treatments became visible on PC2 (Fig. 1D). In contrast, PCoA (Fig. 1C) and hierarchical clustering (Additional file 1: Figure S2) of the bacterial communities recolonizing host tissue revealed a clustering of time points mainly independent of the inocula, even though the beta-diversity distances within time points increased slightly over time (Additional file 1: Figure S6A). Already 2 days post recolonization (dpr), the bacterial communities of all three treatments align to each other and show a high similarity to the bacterial communities of larvae (bL) (Fig. 1C). Interestingly, within the first week of recolonization, the similarity to the bL community increased in all three inocula, while 7 dpr the bacterial communities of all three treatments showed the highest similarity to bL (Fig. 1C, Additional file 1: Figure S7A). Within 2 weeks of recolonization, the bacterial composition of all treatments adjusted to a composition similar to the bacterial composition of juvenile polyps (bJ) (Fig. 1C, Additional file 1: Figure S7B). Twenty-eight dpr, the bacterial communities clustered in between the bacterial communities of juvenile and adult polyps. The recolonization pattern of adult polyps, therefore, reflects the pattern of ontogenetic colonization succession (Fig. 1C, Additional file 1: Figure S7C). This pattern is also evident when calculated with unweighted and weighted UniFrac metrics (Additional file 1: Figure S4). However, this pattern could not be observed during the recolonization of the silicone tubes (Additional file 1: Figure S5, S8).

Analysis of the degree of change of the bacterial communities in the different treatments showed that the bacterial communities of polyps recolonized with bacteria of adult polyps were changed the most (Additional file 1: Figure S6B). In contrast, the bacterial community of animals recolonized with bacteria from larvae exhibited the lowest degree of change (Anosim R = 0.2364, *p* < 0.001; Additional file 1: Figure S6B). However, within 28 dpr the three treatments did not approach the identity of the adult bacterial inoculum completely (Fig. 1C, Additional file 1: Figure S7C). Compared with the wild-type control polyps, which spent the same time in sterile medium without food as the treatment polyps, the treatment polyps approached the wild-type controls after 28 dpr (Additional file 1: Figure S9). This suggests that the difference between the adult microbiota and those recolonized for 28 dpr (Fig. 1C) may be due to starvation.

The comparisons of the alpha diversity also revealed significant qualitative differences. While the bacterial richness increased during the bacterial succession on the host and approached the level of the adult bacterial inoculum after 4 weeks in all treatments (Fig. 1E), the bacterial richness on the tubes remained stable on a low level (Fig. 1F). The changes in absolute bacterial abundance in the two experiments were also opposite. For the recolonization of the host, we measured bacterial abundance via qRT-PCR. For the recolonization of the silicone tubes, we could not use qRT-PCR as it is not possible to normalize to a housekeeping gene. Here, we opted for measuring the biofilm formation with crystal violet. While bacterial abundance on the host tissue decreased over the course of the experiment (Additional file 1: Figure S10A), biofilm formation on the tube increased within the 4-week experimental period (Additional file 1: Figure S10B).

These results suggest that the mechanisms controlling bacterial colonization of host tissue and inert silicone tubes differ significantly. Whereas on the silicone tubes the inocula determined the initial colonization events and thus the subsequent colonization, the initial recolonization events on the host tissue were mainly independent of the inocula. Here, early recolonization events in all treatments were characterized by similar bacterial communities corresponding to the microbiota of early life stages and subsequent recolonization resembled an ontogenetic colonization pattern. Thus, we conclude that the initial recolonization events appear to be strongly influenced by the host but not by the tube, while the subsequent bacteria-bacteria interactions are the main cause of the observed bacterial succession in both host tissue and the tube.

### Recolonization successions resemble ontogenetic colonization sequence

**Fig. 2:**
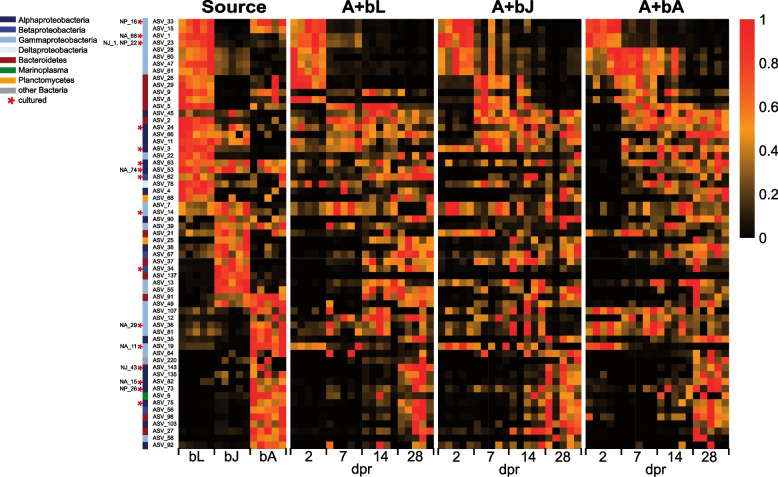
Recolonization dynamics are shown with single ASV level resolution. We selected those ASVs, which were constantly detected in at least one time point across all samples (61 ASVs). The left column represents ASVs present in the microbiome of larvae (bL), juveniles (bJ), and adults (bA), while the next three columns represent the temporal appearance of the 61 ASVs during the recolonization with bacteria isolated from larvae (A + bL), from juveniles (A + bJ), and from adults (A + bA). The five replicates per treatment are shown as separate cells within the columns. The relative abundance of each ASV was scaled between 0 and 1 within each column. Bacterial strains used for the mono-association experiment are marked with the strain name

To determine whether the similarity in polyp recolonization between the three different treatments was due to similar bacterial groups or the same initial colonizers, and to identify bacteria that might act as drivers of the observed bacterial succession, we examined bacterial succession at the exact sequence variant (ASV) level. Therefore, we compared the abundances of the ASVs that are present in at least one of the three inocula with their abundances over the recolonization process (Fig. 2, Additional file 2: Table S3). For this, we filtered for the ASVs with a constant detection during at least one of the four different timepoints of recolonization across all samples (61 ASVs). In the first column of the heatmap, the relative abundance of the selected ASV in the three inocula, bL, bJ and bA, are indicated, illustrating three distinct groups of ASVs characterizing the three different inocula. The three subsequent columns illustrate the relative abundance of these ASVs in the three different recolonization experiments, adult polyps (A) recolonized with bacteria of larvae (+ bL), of juvenile polyps (+ bJ) and adult polyps (+ bA) (Fig. 2).

Interestingly, all three treatments show a similar pattern as seen in the inocula themselves. Two and 7 dpr the most common ASVs are larval-specific in all three treatment (Fig. 2). After 14 dpr, the most common ASVs are specific to larval and juvenile stages, while the relative abundance of some larval-specific ASVs is already decreasing. After 28 dpr, adult-specific ASVs emerge in the community of all three treatments (Fig. 2). Therefore, the most abundant ASVs during the early recolonization process are specific for larval developmental stages, while the most abundant ASVs during the late recolonization are specific for late developmental stages. We conclude from this that the first recolonization events are initiated by larval-specific bacteria and that successively the community composition approaches the identity of the bacterial community of adult polyps. In addition, we infer that these mechanisms are deterministic instead of being dependent on chance for its consistency through all three treatments and replicates.

### Early colonizers show a higher capability of colonization compared to late colonizers

We performed a culturing approach to test the hypothesis that early-colonizing bacteria can readily recolonize *Nematostella*, while late-emerging bacteria only recolonize poorly in mono-association. We isolated 161 bacteria from different developmental stages of *Nematostella* by plating tissue homogenates on three different bacterial media (Additional file 2: Table S1). These isolates belong to a range of Alpha-, Beta-, and Gammaproteobacteria, as well as Actinobacteria (Additional file 2: Table S1). However, we were unsuccessful in culturing Deltaproteobacteria, Bacteroidetes, Planctomycetes, and Spirochaetes.

These isolates were mapped to the ASVs from the recolonization experiment and based on the relative abundance of the ASVs in the heatmap during the recolonization process, they were classified into “early” and “late” colonizers (Fig. 2, Additional file 2: Table S1, S7). If the abundance of an ASV in the inocula did not match the abundance during the recolonization based on the heatmap (Fig. 2), the ASV was assigned based on recolonization pattern. This resulted in a pool of 32 early and 30 late bacterial strains to choose from, however, after correcting for unique ASVs, the pool reduced to 9 early and 6 late strains. We decided on 5 strains each for early and late recolonization by looking for a clear presence in just early or late recolonization in the heatmap (Fig. 2). For early recolonization, we chose two bacterial strains which both correspond to the same ASV (NJ_1 and NP_22).

To test the hypothesis that early colonizers have a higher ability to recolonize adult polyps compared to late colonizers, we performed mono-association experiments. 

**Fig. 3:**
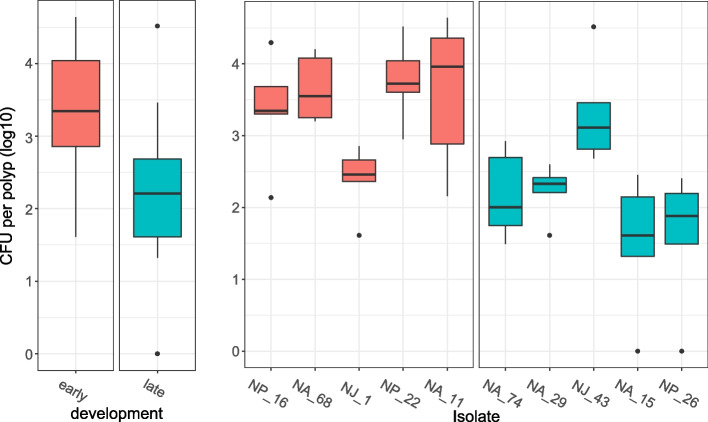
Mono-associations of germfree adult polyps with single bacterial strains. Isolates were classified as early- or late-appearing depending on their appearance during early or late recolonization. Polyps were recolonized with single bacterial isolates for 7 days before polyps were homogenized and spread on MB plates. Colonies were counted after 3 days of incubation (*n* = 5). On the left were all early- or all late-appearing bacteria pooled (Kruskal–Wallis chi-squared = 16.528, *p* < 0.0001). On the right bacteria are shown separately. The *y* axis shows counted colony forming units (CFU) per polyp after recolonization with single bacteria. Data were log10-transformed

While all ten bacterial strains were able to recolonize on germfree polyps, early bacteria colonized *Nematostella* with a significantly higher density than late-appearing bacteria (Kruskal–Wallis chi-squared = 16.528, *p* < 0.0001, Fig. 3). Thus, initial colonization appears to be controlled by the promotion or inhibition of specific bacterial strains, which may be driven by metabolic dependencies or host-controlled mechanisms.

### Metabolic capabilities reflect recolonization pattern

We reconstructed the metabolic networks of bacterial colonizers to estimate the metabolic potential relevant to recolonization and species interactions. The metabolic networks of 31 sequenced isolates (Additional file 2: Table S2, S8) and additional 125 publicly available genomes (Additional file 2: Table S2) were obtained, whereby the selected 16S rRNA genes of the publicly available genomes matched with clusters from the recolonization process by at least 97%. Metabolic networks contain the predicted enzymatic reactions and pathways of an organism and were used to compare metabolic capabilities. We combined the 16S rRNA gene abundance data with predicted metabolic networks to derive potential pathway abundances for each time point during recolonization.

**Fig. 4:**
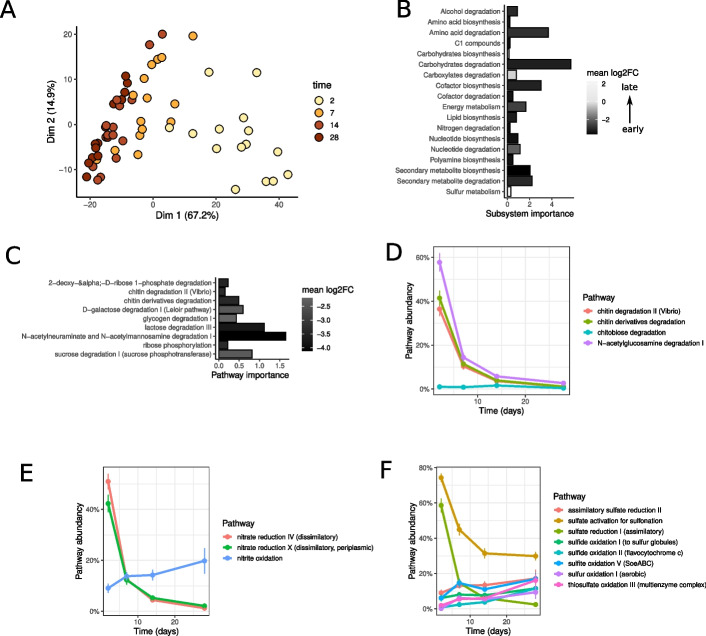
Reconstructions of metabolic networks during bacterial succession. **A** Principal component analysis (PCA) of the metabolic capabilities of the recolonization samples. Each sample contains the metabolic pathway abundances that were derived from inferred metabolic pathways combined with 16S rRNA gene abundance data. Colors indicate the time point of the sample. **B** Pathways associated with early (2d, 7d) and late (14d, 28d) time points were summarized to subsystems. The filling indicates the early vs. late colonizers mean log2 fold change of the pathway abundances at early and late time points. **C** Carbohydrate degradation pathways separating early vs. late colonizers from random forest feature selection. The log2 fold change of mean pathway abundances at early (2d,7d) and late (14d, 28d) time points is shown together with the importance score from random forest feature selection (Boruta). **D** Time series of chitin degradation-associated pathway abundances. The pathway abundance indicates the distribution of pathways among colonizing bacteria (based on 16S relative abundances). Time series of pathway abundances for **E** nitrogen and **F** sulfur cycle-associated pathways

First, we investigated if the bacterial recolonization succession can be found again on the metabolic level. Figure 4A shows the variance of metabolic pathway abundances between samples during colonization as a PCA plot. We found that pathway abundances indeed reflected the observed recolonization pattern, indicated by a separation in dimension 1 of the PCA across time points of recolonization. Pathways that contributed most to dimension 1 were pathways involved in biosynthesis (e.g., cofactor, amino acids, lipids), degradation (e.g., carbohydrates), and energy metabolism (Additional file 2: Table S9). Given the reappearing pattern on the metabolic level, we performed feature extraction by random forests to find pathways associated with early (days = 2,7) and late (days = 14,28) colonizers. We identified 57 pathways reported consistently in repeated feature extractions, and these pathways correctly classified all samples into early or late stages with an accuracy of 93% in k-fold cross-validation.

When pathways were summarized into subsystems, the importance of carbohydrate and amino acid degradation was highest in early colonizers (Fig. 4B). Again carbohydrate degradation showed the most remarkable changes with a mean log2 fold change higher than − 3. Among the identified carbohydrate degradation pathways, polysaccharide degradation (chitin, glycogen) and sugar catabolism (ribose, galactose, lactose, ribose, sucrose) were dominant (Fig. 4C).

Interestingly, we found degradation of chitin and its derivatives among the list of carbohydrate degradation pathways. The pathway abundances of chitin degradation related pathways showed high variance over time, reaching the maximum of 40–60% in early time points (Fig. 4D). The degradation of chitin into monomers of N-acetyl-glucosamine could be accompanied by further utilization. In line with this, the degradation of N-acetyl-glucosamine showed higher abundances also at later time points (Fig. 4D).

In addition, potential bacteria-bacteria interactions involved in bacterial succession were identified by the investigation of complementary pathways and by comparing the changes in their abundances. Nitrate reduction was among the pathways identified by random forest feature selection (subsystem nitrogen degradation in Fig. 4B, Additional file 1: Figure S11). Nitrogen cycling pathways showed a potential link of early nitrate to nitrite reduction with later nitrite oxidation (Fig. 4E). Similarly, feature selection identified hydrogen sulfide oxidation for later colonizers (subsystem sulfur metabolism in Fig. 4B). Moreover, sulfur cycling pathways suggested the early reduction of sulfate followed by later oxidation (Fig. 4F).

### Nematostella shows a common transcriptomic response to bacterial recolonization including chitin synthesis

**Fig. 5:**
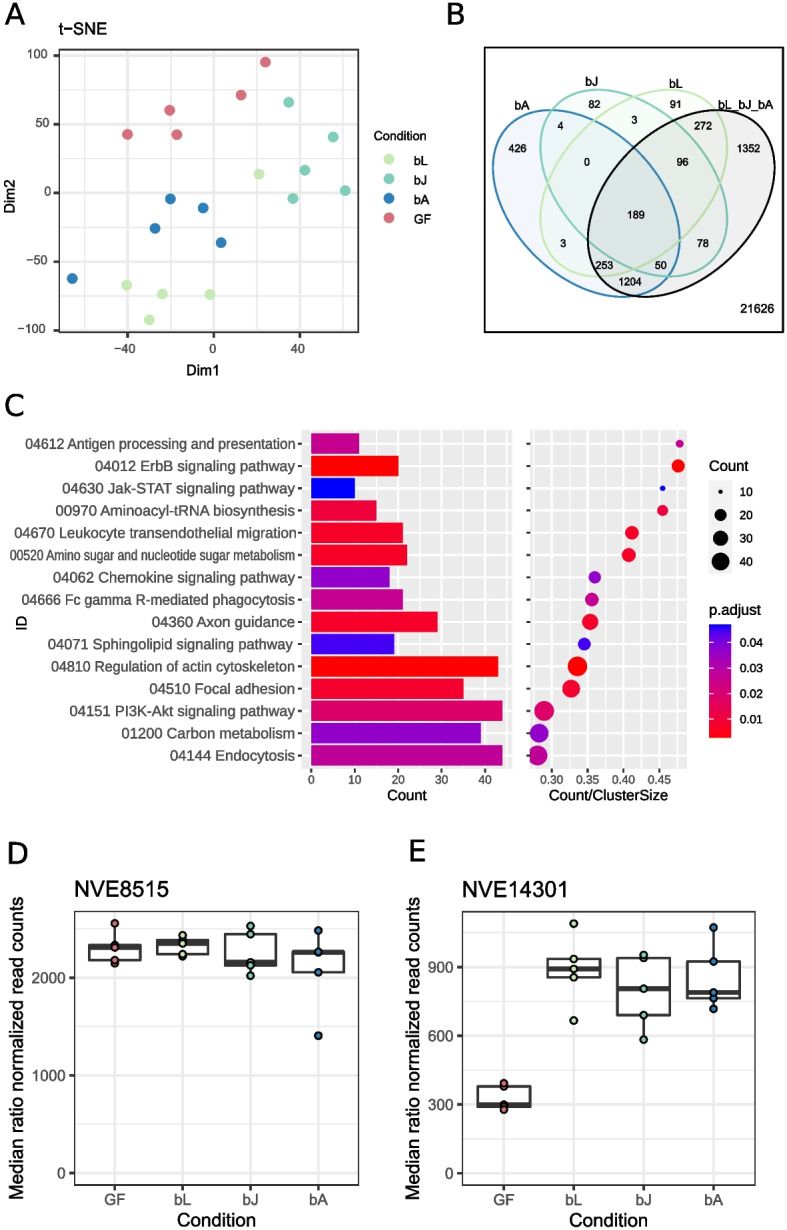
Transcriptomic analysis of recolonized adult polyps 2 days post recolonization. **A** t-SNE plot of the sequenced samples clustering according to their treatment. GF = germfree. **B** Venn diagram of regulated genes in all three treatments and their overlaps. To increase the statistical power, the three comparisons bL vs GF, bJ vs GF, and bA vs GF were separately done to the fourth comparison of bL-bJ-bA vs GF. This way, 1352 more genes could be found that are differentially regulated in all three treatments. **C** Expression of KEGG clusters of all three treatments versus germfree polyps. The barplots show the counts of the genes belonging into the different KEGG clusters, while the dots represent the ratio of the counts to the size of the cluster. **D**, **E** Normalized read counts of the two chitin synthase genes NVE8515 and NVE14301 in *Nematostella*, 2 days post recolonization. **D** Normalized read counts for NVE8515 in recolonized animals compared to germfree animals. **E** Normalized read counts for NVE14301 in recolonized animals compared to germfree animals (log2fc = 2.04, *p* < 0.001)

To identify host mechanisms and functions that might be involved in the selection of early colonizers, we analyzed the common host response to bacterial recolonization. Therefore, we extracted and sequenced the host’s mRNA 2 dpr and compared the response to the three different inocula to each other and to the germfree controls (Fig. 5A). In total, 4103 genes were differentially regulated in recolonized animals in comparison to germfree animals, which represent almost 16% of the whole transcriptome (25,729 genes) (Fig. 5B). Analyzing the host responses to the three inocula, it is notable that animals responded most strongly to the adult inoculum. In total, 2129 genes were differentially regulated in response to the adult inoculum, in contrast to 502 and 907 genes in response to the juvenile and larval inoculum, respectively. This result agrees well with the observation that the microbiota of adult polyps undergoes the greatest change during recolonization (Fig. 1) and that most likely the host is controlling these early colonization events.

With a conventional pairwise analysis, 189 genes could be identified, which were differentially regulated in all three treatments. With a comparison that included a fourth comparison of bL-bJ-bA against GF in addition to the pairwise comparison, we were able to increase this number from 189 to a total of 3494 genes. These 3494 genes are comprised of the 189 originally found genes, 1352 newly discovered genes, and 1953 genes which were found in the pairwise comparisons as exclusively regulated in single comparisons. Therefore, the 3494 genes represent genes that are generally regulated upon contact and eventually colonization by commensal bacteria (Fig. 5B, black circle). From these 3494 regulated genes are 1679 downregulated and 1815 genes upregulated (Additional file 2: Table S10). They belong to a variety of KEGG categories (Fig. 5C). ErbB signaling and Jak-STAT signaling transduce signals through the PI3K-Akt pathway to influence cell proliferation, differentiation, motility, and survival. Fc gamma R-mediated phagocytosis, regulation of actin skeleton, and endocytosis are all involved in engulfment of particles of various sizes. Antigen processing and presentation, leukocyte transendothelial migration, and focal adhesion could indicate a dynamic immune cell response. The enrichment of the KEGG clusters of amino sugar and nucleotide sugar metabolism and carbon metabolism pinpoints toward a mechanism involved in carbohydrate metabolism. Interestingly, among the commonly regulated genes, we found one of the two chitin synthase genes present in *Nematostella* (Fig. 5D, Additional file 2: Table S11). The second chitin synthase was not differentially expressed (Fig. 5E, NVE8515). The gene NVE14301 is upregulated consistently in response to all bacterial recolonization treatments (Fig. 5D) and represents one of the 20 most differentially regulated genes. The coincidence of the high prevalence of chitin degraders among early colonizers and the upregulation of the host’s carbon metabolism, specifically a chitin synthase, suggests that chitin might be essential for the interaction between host and early colonizers.

## Discussion

### Early colonization events are determined by the host

Analyzing the host’s transcriptomic response to the three different bacterial consortia revealed that *Nematostella* reacts strongly to the bacterial recolonization. Remarkably, the strongest response by far was exhibited by the adult polyps that were recolonized with adult bacteria. Looking at the 16S phylogenetic analysis, the community composition resets to a larval-like community, so the adult inoculum must undergo the greatest change. The highest number of regulated genes in these animals suggests that this “reset” is not a bacterial driven process but mainly a host-driven one. This argument is supported by the recolonization of inert silicone tubes. Here, the recolonization succession was mostly dependent on the inoculum and the recolonizations with the three inocula remained separated.

The common response of the polyps to all three inocula suggests how *Nematostella* generally responds to, interacts with, and selects bacterial colonizers. As several pathways regarding phagocytosis are upregulated in combination with signaling pathways involved in cell proliferation and immune cell migration, this suggests that the early transcriptomic response to the recolonization process is a response of the cellular innate immune system. Through its innate immune system, the host can influence and regulate the bacterial communities in a diverse manner. It does not only serve as a defense barrier against pathogens, but also regulates the composition of commensal microbes via, e.g., MyD88-dependent pathways [48, 49] or by the production of AMPs [50, 51]. Either the host shows a broad innate immune response to the bacterial community as a whole, or certain members of the community can elicit a strong immune response. In *Nematostella*, there is growing evidence that nematosomes, small motile multicellular bodies in the gastric cavity, are part of the cellular innate immune system in *Nematostella* [52, 53]. They co-express components of the TLR signaling pathway, as TLR and NF-kB [53], and are able to phagocytose foreign particles and bacteria [52, 54]. As the transcriptomic response to recolonization is dominated by cell proliferation, phagocytosis, and motile immune cell migration, we here further support the hypothesis of nematosomes as part of the innate immune system. Future studies will reveal whether nematosomes, in the form of free-floating immune cell structures, have the ability to selectively phagocytose bacteria and thereby influence colonization of the adult polyp. Differential phagocytosis is already known in the squid-vibrio system, where hemocytes are able to differentiate between the squid’s preferred bacterial symbiont *Vibrio fisheri* and other bacteria of the *Vibrio* genus [55]. In this study, they show that phagocytosis of *V. fisheri* was reduced by pre-exposure of hemocytes to the bacteria, and by the presence of the outer membrane protein OmpU on *V. fisheri*. In a leech model, it is shown that the disruption of the type III secretion system in *Aeromonas veronii* made them vulnerable for phagocytosis by the leech’s macrophage-like cells, while also reducing its pathogenicity in a mouse septicemia model [56].

In regard to the elevated microbial metabolic potential to degrade chitin during the first 2 days, one of the most interesting upregulated host genes is a chitin synthase. Although it has already been known for several years that *Nematostella* possesses at least two genes for chitin synthesis [57], there is just emerging evidence that soft-bodied anemones also express chitin synthase genes [58]. The expression strength indicates a mechanism where chitin is continuously produced while bacteria are present but its production halts if bacteria are missing (Fig. 5E).

In parallel to the increased expression of a chitin-producing enzyme by the host, early-colonizing bacteria had an increased capacity to degrade chitin. Within the metabolic pathways of carbohydrate degradation, chitin degradation differed most between early and late colonizers. The host’s production of chitin, therefore, seemed to be accompanied by microbial utilization.

In general, chitin is widely available in the ocean and chitin degradation activity has been detected for many marine bacteria [59, 60]. In addition, micro-particles of chitin have been shown to enable the community assembly of free-living seawater bacteria [61]. A variety of early-branching metazoans express chitin synthases but it is unknown what function they express [57, 58]. In the context of host-microbiome associations, host-produced chitin is known to modulate the immune response [62]. It has been proposed to enable gut compartmentalization and thus permit barrier immunity from which the mucus layer and its microbial colonization might have been evolved [63]. From this, we concluded that chitin might also play a central role in host-microbiota interactions in *Nematostella* and potentially also in the succession. We hypothesize that host-produced chitin creates a distinct niche that allows chitin-degrading bacteria to flourish and causes the observed succession dynamics.

Commonly, the processes influencing the community assembly can be a combination of deterministic and stochastic processes [9]. Deterministic processes include mechanisms such as the host’s genetic background, its immune system, nutrition, metabolic prerequisites, or environmental factors. Highly deterministic effects are observed in systems such as the *Vibrio* squid system, in which the squid selects *V. fisheri* that is induced to colonize by the production of chemoattractants such as chitobiose and nitric oxide and by attraction via motile cilia [64–66]. Here, the host has complete control over bacterial colonizers. Stochastic processes include priority effects or passive dispersal. In systems where stochasticity is more critical, e.g., due to priority effects, perturbations in microbial composition are observed long during ontogeny, if not into adulthood. Consequently, children born via C-section exhibit a different microbiome than those born vaginally [67], and high levels of hospital pathogens can colonize the infant’s gut, disrupting the transmission of Bacteroides/Bifidobacterium and other commensals [68].

In contrast, our data indicate that chance does not play a significant role in *Nematostella*, as the recolonization dynamics are mainly independent of the inoculum. Similarly, Mortzfeld et al. stated that the developmental age of the host is the main driving force of the *Nematostella* microbiome [14]. However, here we could show that not host ontogeny but host niches and interactions are driving the community composition as we performed experiments on animals which already completed their development.

### Bacterial succession depends mainly on bacteria-bacteria interactions rather than host ontogeny

Once the host has shaped the initial microbial community, microbial forces show a stronger influence on community succession. We observed consistent dynamics up to the establishment of the adult microbiota independent of host ontogeny. Recolonization of adult polyps resulted in a microbial community resembling the community typical of the larval stage, followed by shifts toward a juvenile and adult microbiota. Microbial taxa found at later time points therefore followed a non-random trend. Our mono-association experiments showed higher recolonization success of early-colonizing bacteria compared to late-colonizing bacteria, indicating a mechanism promoting a faster recolonization of early colonizers.

Because the early-colonizing bacteria are not necessarily the bacteria found in large numbers in the adult polyps, they may fit the definition of a keystone species that is present in small numbers but plays a critical role in maintaining community organization and diversity [69]. Especially in ecological systems, keystone species and foundation species are essential for subsequent colonization, e.g., in seaweed forests or in habitats after disturbance [70, 71]. This can imply a niche differentiation or cross feeding events. Datta et al. show that when chitin-covered magnetic beads are submerged in natural marine seawater, the colonization of these beads is mostly determined by the metabolic potential of the bacteria and can be divided into three parts [61]. The first bacteria to settle are specialized in attachment; the second are specialized in metabolizing chitin. The third and last waves of bacteria are specialized in feeding on secondary metabolites of chitin degradation. Similarly, we found chitin followed by chitin derivative degradation for the *Nematostella* microbiome, potentially supporting similar bacteria-bacteria interactions. Interestingly, the alpha diversity over time of these colonized beads showed a similar pattern as the alpha diversity in naturally developing *Nematostella* polyps [14]. The authors hypothesized that this strong drop of alpha diversity shortly after hatching is an effect of metamorphosis and may represent a bottleneck during development. However, the data of Datta et al. and the results presented here are more suggestive of a metabolic bottleneck in the microbiome itself.

Another ambiguity lies in the coherence of the juvenile and the adult microbiome. It is debatable if the juvenile state is an intermediate state before the mature (adult) state is reached, or if it is an alternative state of the microbiome. The uncertainty about that arises firstly from the results of the recolonization, where after 1 month of recolonization, the adult polyps still did not reach the state of the adult inoculum (Fig. 1C), and secondly from the data of wild-type animals that were starved throughout the experiment, whose microbiome converged to the juvenile microbiome over time (Additional file 1: Figure S5). It may be that starvation has a rejuvenating effect on the microbiome. It was shown in several species like fruit flies, rats, and nematodes that caloric or dietary restriction extends the life span by probably downregulating insulin und insulin-like signaling, the amino signaling target of rapamycin (TOR)-S6 kinase pathway, and the glucose signaling Ras-protein kinase A (PKA) pathway [72–74]. The microbiome can also pose a positive influence on longevity by integrating cues from diet which have been shown with a drug-nutrient-microbiome screen [75]. Therefore, we hypothesize that the nutritional status of the polyp influences microbial interactions toward a rejuvenated microbiome and that the observed dynamics during recolonization are also influenced, at least in part, by polyp starvation.

We identified further potential bacteria-bacteria interactions influencing the observed dynamic when investigating metabolic cycles. Early colonizers showed an increased capability to reduce nitrate and sulfate, whereas later colonizing species could oxidize the reduced compounds (nitrite, sulfite, H_2_S). However, as nitrate and sulfate reduction are mostly carried out by anaerobic bacteria, there is an indication that *Nematostella* provides anaerobic niches. In stony corals, which, like *Nematostella*, belong to the class of Hexacorallia, extreme diel fluctuations of oxygen in the vicinity of the polyps are shown, as well as anaerobic nitrate reduction [76, 77]. Oxidation of reduced nitrogen compounds is also a very common process found in coral reefs [78]. Therefore, we propose that interacting reduction–oxidation pathways are important drivers of the bacterial succession dynamics.

## Conclusion

**Fig. 6:**
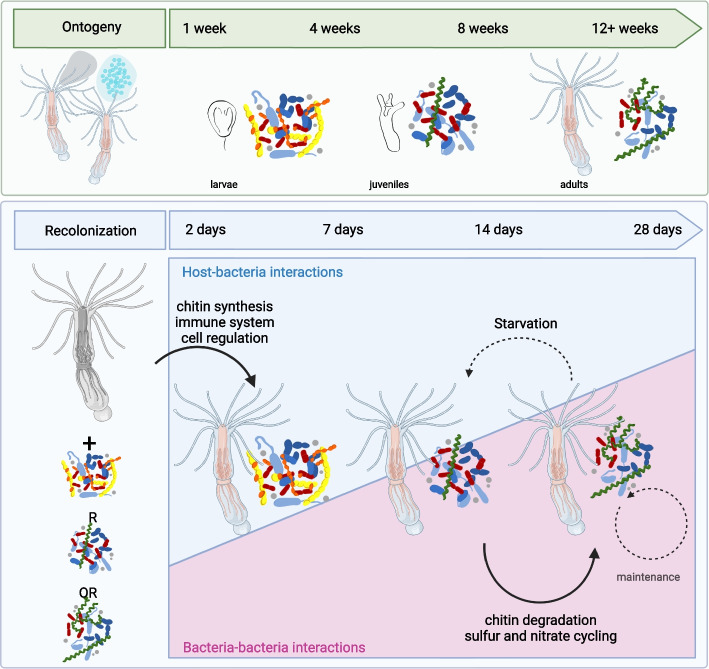
Comparison of the bacterial succession on *Nematostella* during ontogeny and during recolonization. During ontogeny, larvae, juvenile polyps, and adult polyps possess distinct bacterial communities. During recolonization of adult polyps, this bacterial colonization pattern occurring during natural development is recapitulated, independent of the developmental stage from which the bacterial inoculum was isolated. While the bacterial successions during ontogeny take around 3 months, the bacterial successions during recolonization take roughly 4 weeks. While initial selection of bacterial colonizers during recolonization is mainly directed by the host, subsequent bacterial succession, and maintenance are mainly controlled by bacteria-bacteria interactions. Starvation of the host results in a rejuvenation of the microbiome towards a juvenile state. Image created in Biorender

In summary, we uncovered a distinct colonization pattern for the microbiota of *Nematostella* that consistently resulted in very similar bacterial succession in recolonization experiments, regardless of the initial community (Fig. 6).

This colonization pattern recapitulates the colonization pattern occurring during ontogeny, however, in a shorter time frame. As the bacterial successions are independent of the initial community, we conclude that in a marine model system, the establishment of colonization is shaped by the host. Subsequent bacterial succession is mainly determined by bacteria-bacteria interactions, which show a subsequent chitin degradation as well as sulfur and nitrate cycling pathway enrichment during recolonization.

### Supplementary Information

**Additional file 1: Figure S1.** Analysis of the bacterial recolonization dynamics based on 16S upon recolonization of silicone tubes over the course of one month. **Figure S2.** Analysis of the bacterial recolonization dynamics based on 16S upon recolonization of adult polyps over the course of one month. **Figure S3.** Bray Curtis Distance of the recolonization of polyps and tubes together. **Figure S4.** Recolonization dynamics of germfree polyps. **Figure S5.** Recolonization dynamics of silicone tubes. **Figure S6.** Bray–Curtis Dissimilarity Ranks of the bacterial community depending on the time and on the inocula. **Figure S7.** Bray Curtis distance of the bacterial communities on recolonized animals over time in comparison to the inocula. **Figure S8.** Bray Curtis distance of the bacterial communities on recolonized tubes over time in comparison to the inocula. **Figure S9.** Recolonization dynamics of germfree polyps over the course of one month. **Figure S10.** Absolute bacterial load of the polyps (A) and silicone tubes (B) over the course of the recolonization process. **Figure S11.** Predicted metabolic and virulence pathway abundances associated stably with early (2d,7d) and late (14d, 28d) colonizer by random forest feature extraction (Boruta).

**Additional file 2:** **Table S1.** Bacterial strains isolated from Nematostella vectensis with phylogeny according to GenBank Accession number, and ASV names. **Table S2.** Clusters with the genomes (self-sequenced or downloaded from the ncbi database) which were used for the metabolic potential analysis. **Table S3.** Count table for the ASVs for the recolonization of polyps and tubes. **Table S4.** Taxonomy of the ASVs. **Table S5.** Pairwise Permanova on the Bray–Curtis distances for the recolonization of polyps. **Table S6.** Pairwise Permanova on the Bray–Curtis distances for the recolonization of tubes. **Table S7.** ASVs with shortened ASV number and 97% cluster to which they belong. **Table S8.** Bacterial strains from which genomes were sequenced, with developmental stage from which they were isolated, phylogeny and ncbi classification. **Table S9.** Pathways contributing the most to the separation on dimension 1 in the PCA showing the metabolic capabilities during recolonization (Fig. 4A). **Table S10.** Up- and downregulated genes upon recolonization with bacteria, independent of the inoculum (bL-bJ-bA vs GF). **Table S11.** Top20 upregulated genes upon recolonization with bacteria, independent of the inoculum. All relevant scripts can be found on GitHub under https://github.com/Porthmeus/DominEtAl_NematostellaRecolonization and https://github.com/ikmb/assembly-bacteria

## Data Availability

The datasets supporting the conclusions of this article are available in the Sequence Read Archive (SRA) under the accession numbers PRJNA902551 (https://www.ncbi.nlm.nih.gov/bioproject/PRJNA902551) and PRJNA909070 (https://www.ncbi.nlm.nih.gov/bioproject/909070).
